# Inhibitory effect of *ent*-Sauchinone on amyloidogenesis via inhibition of STAT3-mediated NF-κB activation in cultured astrocytes and microglial BV-2 cells

**DOI:** 10.1186/1742-2094-11-118

**Published:** 2014-07-02

**Authors:** Suk-Young Song, Yu Yeon Jung, Chul Ju Hwang, Hee Peom Lee, Chang Hyun Sok, Joo Hwan Kim, Sang Min Lee, Hyun Ok Seo, Byung Kook Hyun, Dong Young Choi, Sang Bae Han, Young Wan Ham, Bang Yeon Hwang, Jin Tae Hong

**Affiliations:** 1 College of Pharmacy and MRC, Chungbuk National University, 52 Naesudong-ro, Heungduk-gu, Cheongju, Chungbuk 361-763, Republic of Korea; 2 College of Pharmacy, Young Nam University, 280 Daehak-Ro, Gyeongsan, Gyeongbuk 712-749, Republic of Korea; 3 Department of Chemistry and Biochemistry, Brigham Young University, C409 BNSN, 84602 Provo, UT, USA

**Keywords:** *ent*-Sauchinone, nuclear factor-kappa B, neuroinflammation, STAT3

## Abstract

**Background:**

*ent*-Sauchinone is a polyphenolic compound found in plants belonging to the lignan family. *ent*-Sauchinone has been shown to modulate the expression of inflammatory factors through the nuclear factor-kappa B (NF-κB) signaling pathway. It is well known that neuroinflammation is associated with amyloidogenesis. Thus, in the present study, we investigated whether *ent*-Sauchinone could have anti-amyloidogenic effects through the inhibition of NF-κB pathways via its anti-inflammatory property.

**Methods:**

To investigate the potential effect of *ent*-Sauchinone on anti-neuroinflammation and anti-amyloidogenesis in *in vitro* studies, we used microglial BV-2 cells and cultured astrocytes treated with *ent*-Sauchinone (1, 5, and 10 μM) for 24 hours. For the detection of anti-neuro-inflammatory responses, reative oxygen species (ROS) and Nitric oxide (NO) generation and inducible nitric oxide synthase (iNOS) and cyclooxygenase-2 (COX-2) expression were measured with assay kits and western blotting. β-secretase and β-secretase activities and β-amyloid levels were determined for measuring the anti-amyloidogenic effects of *ent*-Sauchinone by enzyme assay kits. NF-κB and STAT3 signals were detected with electromobility shift assay (EMSA) to study the related signaling pathways. The binding of *ent*-Sauchinone to STAT3 was evaluated by a pull-down assay and by a docking model using Autodock VINA software (Hoover’s Inc., Texas, United states).

**Results:**

*ent*-Sauchinone (1, 5, and 10 μM) effectively decreased lipopolysaccharide (LPS)-(1 μg/ml) induced inflammatory responses through the reduction of ROS and NO generations and iNOS and COX-2 expressions in cultured astrocytes and microglial BV-2 cells. *ent*-Sauchinone also inhibited LPS-induced amyloidogenesis through the inhibition of β-secretase and β-secretase activity. NF- κB amyloid and STAT3, critical transcriptional factors regulating not only inflammation but also amyloidogenesis, were also inhibited in a concentration dependent manner by *ent*-Sauchinone by blocking the phosphorylation of I κB and STAT3 in cultured astrocytes and microglial BV-2 cells. The docking model approach showed that *ent*-Sauchinone binds to STAT3, and the employment of a STAT3 inhibitor and siRNA reversed *ent-*Sauchinone-induced inhibition NF-κB activation and Aβ generation.

**Conclusions:**

These results indicated that *ent*-Sauchinone inhibited neuroinflammation and amyloidogenesis through the inhibition of STAT3-mediated NF-κB activity, and thus could be applied in the treatment of neuro-inflammatory diseases, including Alzheimer’s disease.

## Background

Alzheimer’s disease (AD) is an age-related neurodegenerative disease characterized by the accumulation of beta amyloid (A), an insoluble peptide deposited extracellularly in the brain, causing senile plaques [1]. This hydrophobic polypeptide is the product of proteolytic cleavage of the amyloid precursor protein (APP). Brains of AD patients exhibit a number of pathological abnormalities including a profound loss of synapses, microglial activation, and inflammatory processes [2]. Inflammatory reactions and mediators have been reported to augment APP expression and A formation [3,4] and transcriptionally up-regulate mRNA, protein levels, and enzymatic activity of β-secretase, a key enzyme in the production of A [5]. Recently, we and other researchers have also shown that anti-inflammatory agents prevent A deposition [6,7] and that anti-inflammatory agents prevent A deposition in cultured neuronal cells [7-9] as well as in mouse models of AD [7,10]. Moreover, McGeer *et al*. suggested that anti-inflammatory agents could be applicable for the treatment of patients with AD [11]. These observations strongly suggest that anti-inflammatory agents could be effective for the prevention of AD prevalence through the reduction of A generation and/or deposition [12].

In most neurodegenerative disorders, massive neuronal cell death occurs as a consequence of an uncontrolled neuro-inflammatory response by the activation of astrocytes and microglia [13]. Glial cells such as astrocytes and microglia can induce cytokines, reactive oxygen radicals (ROS), and nitric oxide (NO) which lead to exaggeration of the disease processes [14]. Expression of inducible nitric oxide synthase (iNOS) and cyclooxygenase-2 (COX-2) can be regulated by the activation of nuclear factor-κB (NF-κB) because there is one NF-κB DNA consensus sequence within the COX-2 promoter [15], and two NF-κB DNA consensus sequences within the iNOS promoter [16]. Moreover, NF-κB DNA consensus sequences are also located in the promoter of neuronal β-secretase (BACE 1). Thus, dysregulation of NF-κB would provide a potential approach for the management of AD through the reduction of both neuroinflammation and A generation [17]. Signal transducer and activator of transcription 3 (STAT3) is also a significant regulator of neuroinflammation and A generation [18]. We demonstrated that inactivation of STAT3 inhibited A generation and neuroinflammation through the suppression of NF-κB activation. [19]. Sauchinone is reported to perform a variety of biological activities such as hepatoprotective, anti-inflammatory actions and inhibitory effects on bone resorption [20]. However, it is not clear yet whether *ent*-Sauchinone could show an anti-neuroinflammatory response and thus an anti-amyloidogenic effect. In the present study, we investigated anti-neuroinflammatory and anti-amyloidogenic activities of *ent*-Sauchinone and its possible mechanisms in cultured BV-2 cells and astrocytes.

## Materials and methods

### Chemicals and reagents

Cell culture mediums and agents such as Dulbecco’s Modified Eagle’s Medium (DMEM, Invitrogen, Carlsbad, California, United States), fetal bovine serum (FBS), penicillin, streptomycin, Eagle’s salts, L-glutamine, pyruvate, potassium chloride (KCl), and antibiotics were purchased from Invitrogen (Carlsbad, California, United States) and trypsin was purchased from Sigma-Aldrich (St Louis, Missouri, United States). Opti-MEM medium, TRIzol reagent, Lipofectamine, and geneticin (G418) were also acquired from Invitrogen (Carlsbad, California, United States). siRNA and specific antibodies (rabbit polyclonal antibodies against p65 and I B and mouse monoclonal antibody against p50) were obtained from Santa Cruz Biotechnology Inc. (Santa Cruz, California, United States). Rabbit polyclonal antibodies against iNOS and COX-2 were obtained from Cayman Chemical (1:1,000, Ann Arbor, Michigan, United States). The gel shift assay system was purchased from Promega (Madison, Wisconsin, United States). The -secretase activity kit was provided from Abcam, Inc. (Cambridge, Massachusetts, United States). An ELISA kit for A _1-42_ level determination was purchased from Immuno-Biological Laboratories Co., Ltd. (Tokyo, Japan). Cyanogen bromide Epoxy-activated Sepharose 6B and all other chemicals were obtained from Sigma-Aldrich (St Louis, Missouri, United States).

### Preparation of *ent*-Sauchinone

**Figure 1:**
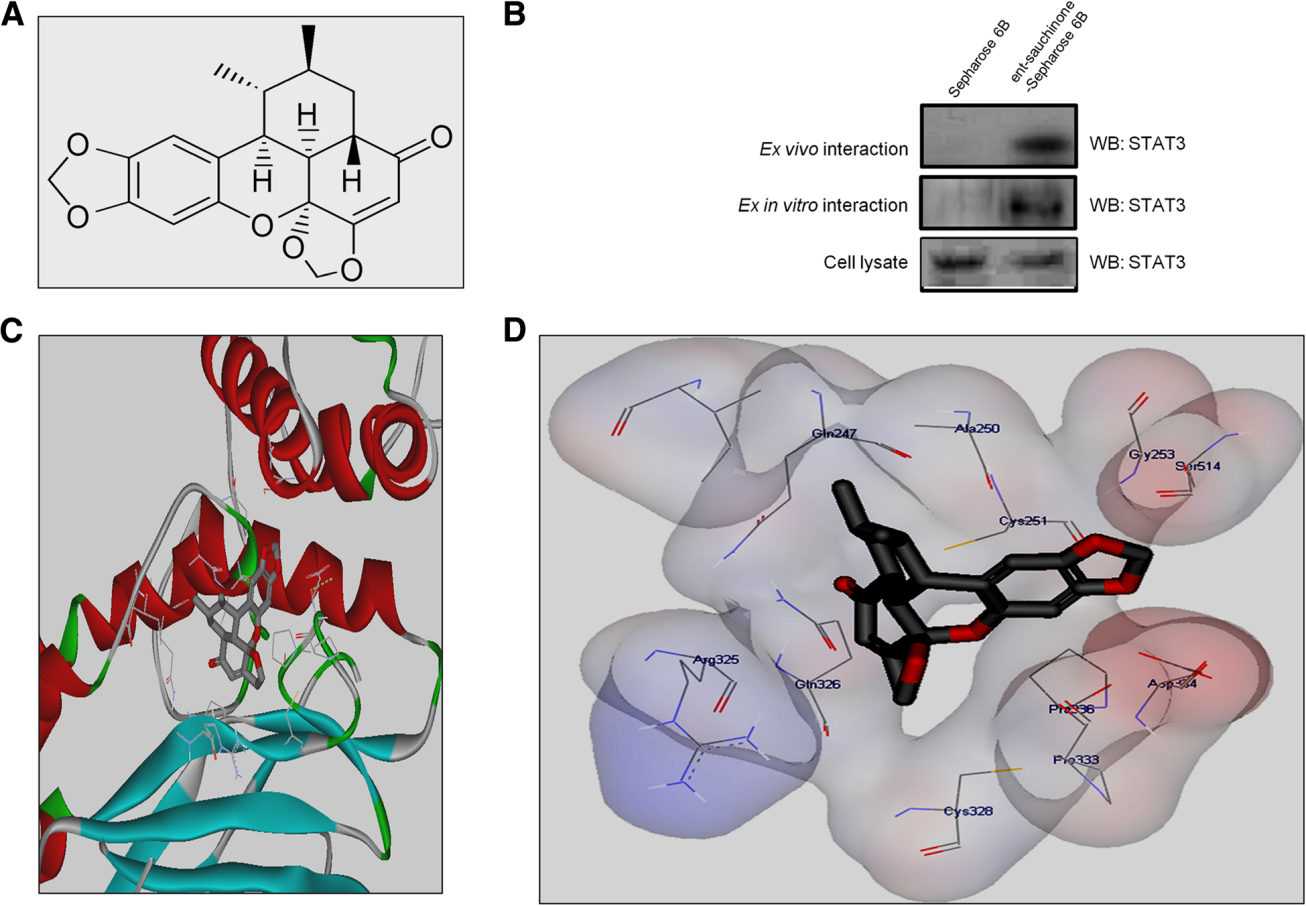
**Structural interaction between *ent*-Sauchinone and STAT3.***ent*-Sauchinone was prepared from 4-(oxiran-2-yl) phenyl acetate, Pd(OAc)2 and tributyl phosphine **(A)**. Pull-down assay identifies an interaction between the *ent*-Sauchinone and STAT3. *ent*-Sauchinone was conjugated with cyanogen bromide Epoxy-activated Sepharose 6B **(B)**. Docking model of *ent*-Sauchinone with STAT3 as described in Materials and Methods **(C and D).**

The aerial parts of *Saururus chinensis* were purchased from Kyungdong Oriental Herbal Market in Seoul, Korea, September 2011 and identified by one of the authors (BYH). A voucher specimen (CBNU-SC-2011) has been deposited at the Herbarium of College of Pharmacy, Chungbuk National University, Korea. The aerial parts of *S. chinensis* (5.0 kg) were extracted with MeOH three times (18 L × 3, overnight). The filtrate was evaporated under reduced pressure to obtain a MeOH extract (540 g), which was suspended in distilled water and partitioned with n-hexane (40 g) and CH_2_Cl_2_ (100 g). The CH_2_CI_2_-soluble fraction (100 g) was separated over a silica gel column with a n-hexane-CH_2_CI_2_-MeOH gradient to yield 20 fractions (SCC01-SCC20). Fraction SCC-6 (0.5 g) was purified on a Sephadex LH-20 (CH_2_Cl_2_-MeOH, 1:1) and preparative HPLC (Waters system, YMC (YMC CO., LTD., Kyoto, Japen) ODS (octadecyl silane) H-80 column, 150 × 20 mm i.d (internal diameter), MeCN:H_2_O gradient, from 50:50 to 90:10, flow rate 6 mL/min) to obtain *ent*-Sauchinone (50 mg). The structure of *ent*-Sauchinone (Figure 1A) was determined by the comparison of its physicochemical and spectroscopic data as reported previously [20]. *ent*-Sauchinone: Colorless needle crystal; [α]^25^_D_ -110.8^o^; ESI-MS m/z 379 [M + Na]^+^; ^1^H-NMR (CDCl_3_, 500 MHz) δ_H_ 6.86 (1H, s, H-6), 6.45 (1H, s, H-3), 5.95 (1H, br d, *J* = 1.5 Hz, H-10a), 5.91 (1H, br d, *J* = 1.5 Hz, H-10b), 5.69 (1H, s, H-10’a), 5.64 (1H, s, H-10’b), 5.54 (1H, s, H-3’), 3.07 (1H, d, *J* = 5.3 Hz, H-7), 2.54 (1H, m, H-1’), 2.52 (1H, d, *J* = 5.3 Hz, H-6’), 2.48 (1H, m, H-8), 1.94 (1H, dt, *J* = 13.0, 3.4 Hz, H-7’a), 1.66 (1H, m, H-7’b), 1.92 (1H, m, H-8’), 1.23 (3H, d, *J* = 7.2 Hz, H-9), 0.75 (3H, d, *J* = 7.5 Hz, H-9’); ^13^C-NMR (CDCl_3_, 125 MHz) δ _C_ 115.6 (C-1), 144.9 (C-2), 99.1 (C-3), 143.1 (C-4), 146.6 (C-5), 106.4 (C-6), 34.9 (C-7), 34.7 (C-8), 21.2 (C-9), 37.4 (C-1’), 199.5 (C-2’), 100.1 (C-3’), 168.5 (C-4’), 100.3 (C-5’), 37.5 (C-6’), 25.1 (C-7’), 33.3 (C-8’), 20.8 (C-9’), 101.2 (OCH_2_O), 98.5 (OCH_2_O).

### BV-2 cell culture

BV-2 cells were obtained from the American Type Culture Collection (Rockville, Maryland, United States). These cells were maintained at subconfluence in a 95% air/5% CO_2_ humidified atmosphere at 37°C. The medium used for routine subcultivation was DMEM (Invitrogen, Carlsbad, California, United States), supplemented with 10% FBS, penicillin (100 units/ml), and streptomycin (100 μg/ml). Cells were counted with a hemocytometer and the number of viable cells was determined through trypan blue dye exclusion.

### Astrocyte culture

Sprague-Dawley rats were maintained in accordance with the policy of the National Institute of Toxicological Research, which is in accordance with the Korea Food and Drug Administration’s guideline for the care and use of laboratory animals. The Sprague-Dawley rats weighing between 200 and 300 g were housed under 12 hour light/dark cycles, at 23°C and 60 ± 5% humidity. All animals had free access to food (Samyang Foods, Seoul, Korea) and water. Cerebral cortical cells were isolated from the neonatal rat brains (day 1) in PBS (0.1 mol). After washing with DMEM, the isolated cells were incubated for 15 minutes in DMEM containing 0.2% trypsin. Cells were dissociated by trituration and plated into polyethyleneimine-coated plastic (5 × 10^5^ cells/60 mm dish) containing minimum essential medium with Eagle’s salts supplemented with 10% heat-inactivated FBS, 2 mM L-glutamine, 1 mM pyruvate, 20 mM KCl, 10 mM sodium bicarbonate, and 1 mM Hepes (pH 7.2). After three days in culture, the culture medium was replaced with DMEM containing 10% FBS. The culture medium was changed every three days. Microglia and oligodendrocytes were isolated after the 12-day culture by shaking off, then further incubated with new DMEM with 10% FBS. The astrocytes were judged by cell morphology and by immunostaining with antibodies against glial fibrillary acidic protein (GFAP). Cells were fixed in 4% paraformaldehyde, then incubated with the antibody; GFAP (1:200 Cell Signaling Technology, Inc., Beverly, Massachusetts, United states), and visualized using Alexa Fluor-conjugated secondary antibody (Life Technologies, Seoul, Korea). Nuclei were stained with 4′,6-diamidine-2-phenylindole dihydrochloride. Of the cells in the astrocyte cultures, 95% were GFAP-positive. The astrocytes were more than 95%. The astrocytes were incubated in the DMEM media with 10% FBS during treatment. Cells grown on LabTek chamber slides (Nalge Nunc International, New York, United States) were used for immunochemical studies.

### Transfection

The cultured cells were treated simultaneously with LPS (1 g/ml) and several concentrations (1, 5, and 10 μM) of *ent*-Sauchinone in 0.05% dimethyl sulfoxide (DMSO), and the cells were harvested after 24 hours. For transient transfection, cells were plated in 100-mm plates and transiently transfected with STAT3 siRNA (Santa Cruz Biotechnology Inc.) using the WelFect-EX PLUS reagent in OPTI-MEN, according to the manufacturer’s specification (WelGENE, Seoul, Korea).

### Cell viability assay

The cytotoxicity of *ent*-Sauchinone was evaluated using a WST-8 assay (Dojindo Laboratories, Tokyo, Japan). WST-8 [2-(2-methoxy-4-nitrophenyl)-3(4-nitro-phenyl)-5-(2,4-disulfo-phenyl)-2H-tetrazolium, monosodium salt] is reduced by de-hydro-genases in cells to give a yellow-colored product (formazan), which is soluble in the culture medium. The amount of the formazan dye generated by the activity of dehydro-genases in cells is directly proportional to the number of living cells. In brief, 1 × 10^4^ cells (astrocytes and microglial BV-2 cells) per well were plated into 96-well plates, incubated at 37°C for 24 hours, and given a fresh change of medium. Cells were then incubated in the DMEM media containing 10% FBS with or without LPS (1 μg/ml) in the absence or presence of various concentrations of *ent*-Sauchinone at 37°C for an additional 24 hours. At that point, 10 μl of the WST-8 solution was added to the wells and incubation continued for another hour. The resulting color was assayed at 450 nm using a microplate absorbance reader (Sunrise^®^, TECAN, Männedorf, Switzerland).

### Determination of NO production

Cells were grown in 96-well plates and then incubated with or without LPS (1 μg/ml) in the absence or presence of various concentrations of *ent*-Sauchinone for 24 hours. The nitrite accumulation in the supernatant was assessed by the Griess reaction [21]. Each 50 μl of culture supernatant was mixed with an equal volume of Griess reagent [0.1% N-(1-naphthyl)-ethylenediamine, 1% sulfanilamide in 5% phosphoric acid] and incubated at room temperature for 10 minutes. The absorbance at 540 nm was measured in a microplate absorbance reader and a series of known concentrations of sodium nitrite was used as a standard.

### Measurement of ROS

Generation of ROS was assessed by 2,7-dichlorofluorescein diacetate (DCFH-DA, Sigma-Aldrich), an oxidation sensitive fluorescent probe. Intracellular H_2_O_2_ or low molecular-weight peroxides can oxidize 2,7-dichlorofluorescein diacetate to the highly fluorescent compound dichlorofluorescein (DCF). Briefly, astrocytes and microglial cells were plated in 6-well plates (5 × 10^5^), and subconfluent cells were subsequently treated with *ent*-Sauchinone (1, 5, and 10 μM) for 30 minutes. After the cells were trypsinized, 1 × 10^4^ cells were plated in a black 96-well plate and incubated with 10 μM DCFH-DA at 37°C for 4 hours. Fluorescence intensity of DCF was measured in a microplate-reader at an excitation wavelength of 485 nm and an emission wavelength of 538 nm.

### Western blot analysis

Cells were homogenized with Protein Extraction Solution (PRO-PREP^®^, Intron Biotechnology, Seongnam, Korea), and lysed for 40 minutes incubation on ice. The lysate centrifuged at 15,000 rpm for 15 minutes. Equal amount of proteins (40 μg) were separated on a SDS/10%-polyacrylamide gel, and then transferred to a polyvinylidene difluoride (PVDF) membrane (GE Water and Process technologies, Trevose, Pennsylvania, United States). Blots were blocked for 2 hours at room temperature with 5% (w/v) non-fat dried milk in Tris-buffered saline tween-20 (TBST: 10 mM Tris (pH 8.0) and 150 mM NaCl solution containing 0.05% tween-20). After a short wash in TBST the membrane was incubated at room temperature with specific antibodies. Rabbit polyclonal antibodies against iNOS and COX-2 (1:1,000) (Cayman Chemical, Ann Arbor, Michigan, United States), rabbit polyclonal antibodies against p65 and IκB (1:500), and mouse monoclonal antibody against p50 (1:500) (Santa Cruz Biotechnology Inc. Santa Cruz, California, United States) were used in the study. The blot was then incubated with the corresponding conjugated anti-rabbit or mouse immunoglobulin G-horseradish peroxidase (Santa Cruz Biotechnology Inc.). Immunoreactive proteins were detected with the enhanced chemiluminescence (ECL) western blotting detection system.

### Gel electromobility shift assay (EMSA)

Gel shift assays were performed according to the manufacturer’s recommendations (Promega, Madison, Wisconsin, United States). Briefly, 5 × 10^6^ cells were washed twice with 1 × PBS, followed by the addition of 1 ml of PBS, and then the cells were scraped into a cold Eppendorf tube. Cells were spun down at 13,000 rpm for 5 minutes and the resulting supernatant was removed. Cells were suspended in 400 μl of solution A containing 10 mM HEPES, pH 7.9, 1.5 mM MgCl_2_, 10 mM KCl, 0.5 mM dithiothreitol, and 0.2 mM phenylmethylsulfonyl fluoride; vigorously vortexed; allowed to incubate on ice for 10 minutes; and centrifuged at 12,000 rpm for 6 minutes. The pelleted nuclei were resuspended in solution C (solution A + 420 mM NaCl, 20% glycerol) and allowed to incubate on ice for 20 minutes. The cells were centrifuged at 15,000 rpm for 15 minutes and the resulting nuclear extract supernatant was collected in a chilled Eppendorf tube. Consensus oligonucleotides were end-labeled using T4 polynucleotide kinase and [γ-^32^P] adenosine triphosphate (ATP) for 10 minutes at 37°C. Gel shift reactions were assembled and allowed to incubate at room temperature for 10 minutes followed by the addition of 1 μl (50,000 to 200,000 cpm (Count per minute) of ^32^P end-labeled oligonucleotide, followed by another 20 minutes of incubation at room temperature. Subsequently, 1 μl of gel loading buffer was added to each reaction and loaded onto a 6% non-denaturing gel and electrophoresis until the dye was four-fifths of the way down the gel. The gel was dried at 80°C for 1 hour and exposed to film overnight at -70°C.

### Secretase activities assay

β-secretase activity in BV-2 cells and astrocytes were determined using a commercially available β-secretase activity kit (Abcam, Inc, Cambridge, Massachusetts, United States). Protein was extracted from cells using an ice-cold extraction buffer, incubated on ice for 20 minutes and centrifuged at 10,000 rpm for 5 minutes at 4°C. The supernatant was collected. A total of 50 μL of sample (total protein 100 μg) was added to each well followed by 50 μL of 2 × reaction buffer and 2 μL of β-secretase substrate incubated in the dark at 37°C for 2 hours. Fluorescence was read at excitation and emission wavelengths of 355 and 510 nm respectively, using a Fluostar Galaxy fluorometer (BMG Lab Technologies, Offenburg, Germany) with Felix software (BMG Lab Technologies, Offenburg, Germany). β-secretase activity is proportional to the fluorimetric reaction, and is expressed as nmol/mg protein per minute.

### Fluorescence microscopy

The fixed cells were exposed to the following primary antibodies; GFAP, Iba1 and Aβ_1-42_ (1: 100 dilutions in blocking serum, Cell Signaling Technology, Inc., Beverly, Massachusetts, United States) at room temperature for 1 hour. After incubation, the cells were washed twice with ice-cold PBS and incubated with an anti-rabbit or mouse secondary antibody conjugated to Alexa Fluor 488 or 568 (Invitrogen-Molecular Probes, Carlsbad, California, United States) at room temperature for 1 hour. Immunofluorescence images were acquired using an inverted fluorescent microscope Zeiss Axiovert 200 M (Carl Zeiss, Thornwood, New York, United States).

### Measurement of Aβ levels

Cell lysates (the same preparation of lysates as used for western blotting) were obtained using a protein extraction buffer containing protease inhibitor, 4-(2-aminoethyl)-benzene sulfonyl fluoride. Aβ_1-42_ levels were determined using specific ELISAs (IBL, Immuno-Biological Laboratories Co., Ltd., Fujioka, Japan). In brief, 100 μl of sample was added to precoated plates and was incubated overnight at 4°C. After washing each well of the precoated plate with washing buffer, 100 μl of labeled antibody solution was added and the mixture was incubated for 1 hour at 4°C in the dark. After washing, chromogen was added and the mixture was incubated for 30 minutes at room temperature in dark. Finally, the resulting color was assayed at 450 nm using a microplate absorbance reader (Sunrise^®^, TECAN, Männedorf, Switzerland) after the addition of stop solution.

### Pull-down assay

*ent*-Sauchinone was conjugated with cyanogen bromide Epoxy-activated Sepharose 6B (Sigma, St Louis, Missouri, United States). Briefly, *ent*-Sauchinone (1 mg) was dissolved in 1 ml of coupling buffer (0.1 M NaHCO3 and 0.5 M NaCl, pH 6.0). The Epoxy-activated Sepharose 6B was swelled and washed in 1 mM HCl on a sintered glass filter, then washed with a coupling buffer. Epoxy-activated Sepharose 6B beads were added to the *ent*-Sauchinone containing coupling buffer and incubated at 4°C for 24 hours. The *ent*-Sauchinone-conjugated Sepharose 6B was washed with three cycles of alternating pH wash buffers (buffer 1: 0.1 M acetate and 0.5 M NaCl, pH 4.0; buffer 2: 0.1 M TriseHCl and 0.5 M NaCl, pH 8.0). *ent*-Sauchinone-conjugated beads were then equilibrated with a binding buffer (0.05 M TriseHCl and 0.15 M NaCl, pH 7.5). The control unconjugated Epoxy-activated Sepharose 6B beads were prepared as described above with the absence of *ent*-Sauchinone. The cell lysate or STAT3 recombinant protein (Abnova, Taipei, Taiwan) were mixed with *ent*-Sauchinone conjugated Sepharose 6B or Sepharose 6B at 4 C for 24 hours. The beads were then washed three times with TBST. The bound proteins were eluted with SDS loading buffer. The proteins were then resolved by SDS-PAGE followed by immunoblotting with antibodies against STAT3 (1:1000 dilution, Santa Cruz Biotechnology Inc.).

### Molecular modeling

Docking studies between STAT3 and *ent*-Sauchinone were performed using Autodock VINA [22]. STAT3 was obtained from the X-ray crystal structure of dimeric unphosphorylated STAT3 core fragment (PDB (Protein Data Bank) ID: 3CWG) [23]. Only one monomer of the homo-dimeric STAT3 crystal structure was used in the docking experiments and conditioned using AutodockTools by adding all polar hydrogen atoms. A three dimensional structure of *ent*-Sauchinone was built using the ChemBio3D (PerkinElmer Informatics, Massachuserrs, United states) and Discovery Studio 3.5 Client (Accelrys, Inc., California, United states), which was further prepared using AutodockTools. The grid box was centered on the STAT3 monomer and the size of the grid box was adjusted to include the whole monomer. Docking experiments were performed at various exhaustiveness values of the default, 16, 24, 32, 40, and 60.

### Statistical evaluation

The data represent the mean ± S.E. of three independent experiments performed in triplicate. Statistical analysis was performed by one-way analysis of variance (ANOVA), followed by a Dunnett test as a *post hoc* comparison. Differences were considered significant at *P* <0.05.

## Results

### Effect of *ent*-Sauchinone on astrocyte and microglial BV-2 cell viability

Co-treatment with (1, 5, and 10 μM) *ent*-Sauchinone and 1 μg/ml LPS resulted in a slight increase in the cell viability of astrocytes (Additional file 1: Figure S1A) and microglial BV-2 cells (Additional file 1: Figure S1B). Thus, in this study, anti-inflammatory and anti-amyloidogenesis effects were active with up to 10 μM of *ent*-Sauchinone.

### Effect of *ent*-Sauchinone on LPS-induced ROS and NO production, and iNOS and COX-2 expression in BV-2 cells and astrocytes

**Figure 2:**
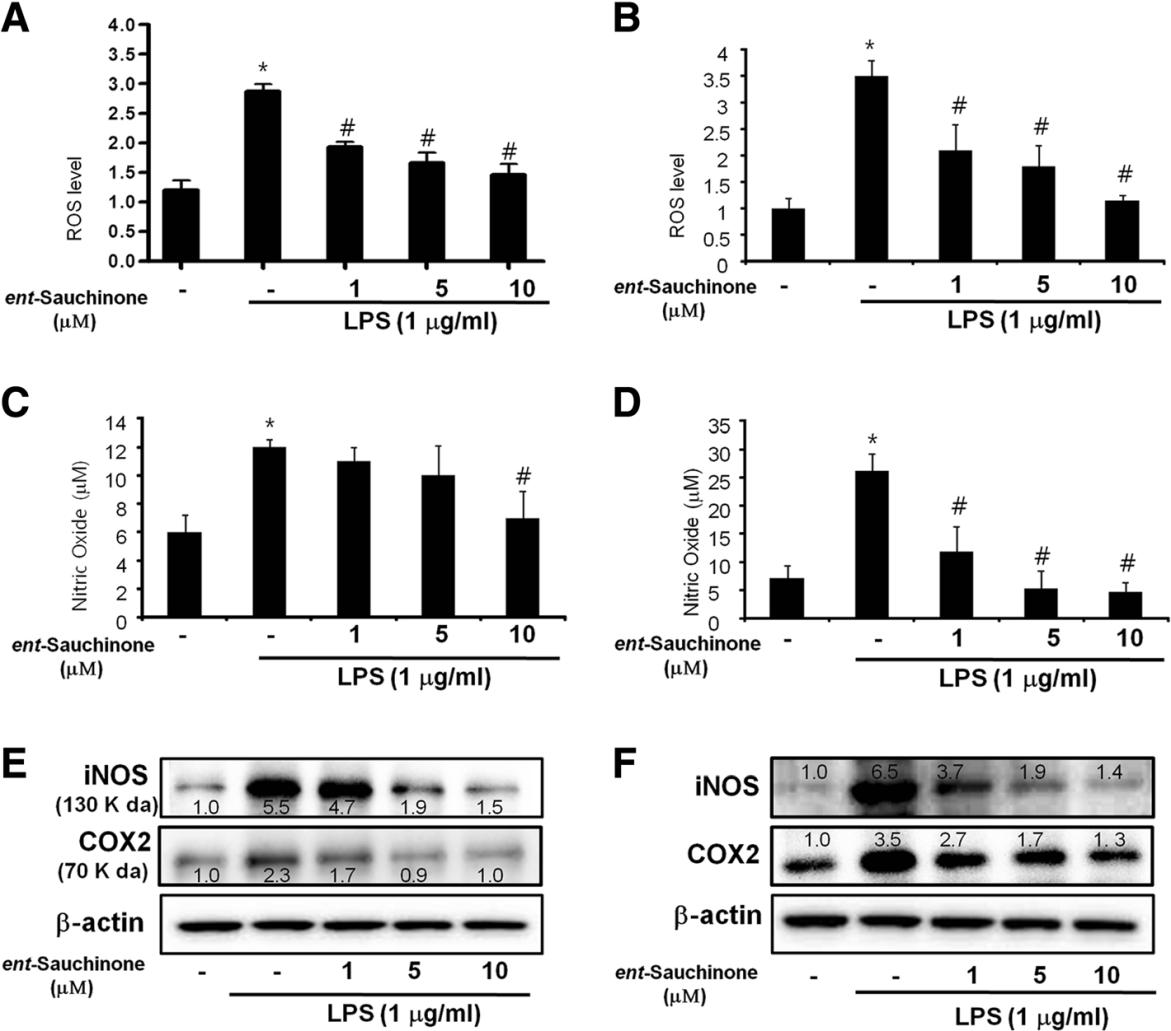
**Effect of *ent*-Sauchinone on LPS-induced ROS and NO release, protein expressions of iNOS and COX-2, and in astrocytes and microglial BV-2 cells.** Cells were treated with 1 μg/ml of LPS alone, or with LPS plus different concentrations (1, 5, and 10 μM) of *ent*-Sauchinone, at 37°C for 24 hours. Intracellular ROS levels were determined by measuring DCF fluorescence **(A and B)**. NO level was determined by Griess reaction, as described in Materials and Methods, in supernatants from astrocytes **(C)** and microglial BV-2 cells **(D)**. Astrocytes **(E)** and microglial BV-2 cells **(F)** were treated with 1 μg/ml of LPS alone, or with LPS plus different concentrations (1, 5, and 10 μM) of *ent*-Sauchinone, at 37°C for 24 hours. Equal amounts of total protein (40 μg/lane) were subjected to 10% SDS-PAGE, and the expression of iNOS and COX-2 were detected by western blotting using specific antibodies. β-Actin protein was used here as an internal control. *indicates significantly different from the control group (*P* <0.05.. ^#^indicates significantly different from the LPS-treated group (*P* <0.05.. Values below or above of each figures mean quantified relative expression of the proteins.

To study the protective effect of *ent*-Sauchinone on LPS-induced activation of astrocytes and microglial BV-2 cells, the cells were treated with or without *ent*-Sauchinone in the presence of LPS (1 μg/ml). Release of ROS and NO was determined as an indicator of astrocyte and microglial BV-2 cell activation as well as oxidative stress. We also found that co-treatment of *ent*-Sauchinone reduced LPS- (1 μg/ml) induced ROS in astrocytes (Figure 2A) and in microglial BV-2 cells (Figure 2B), as well as NO generation in astrocytes (Figure 2C) and in microglial BV-2 cells (Figure 2D). iNOS and COX-2 expression was then determined by western blot analysis since iNOS can be also modulated by COX-2. As shown in Figure 2E and F, expression of iNOS and COX-2 was significantly lowered in an unstimulated condition. However, iNOS and COX-2 expression was markedly increased in response to LPS (1 μg/ml) after 24 hours. However, co-treatment with *ent*-Sauchinone (1, 5, and 10 μM) caused a concentration-dependent decrease of iNOS and COX-2 expression in astrocytes (Figure 2E) and BV-2 cells (Figure 2F).

### Effect of *ent*-Sauchinone on LPS-induced NF-κB DNA binding activities

**Figure 3:**
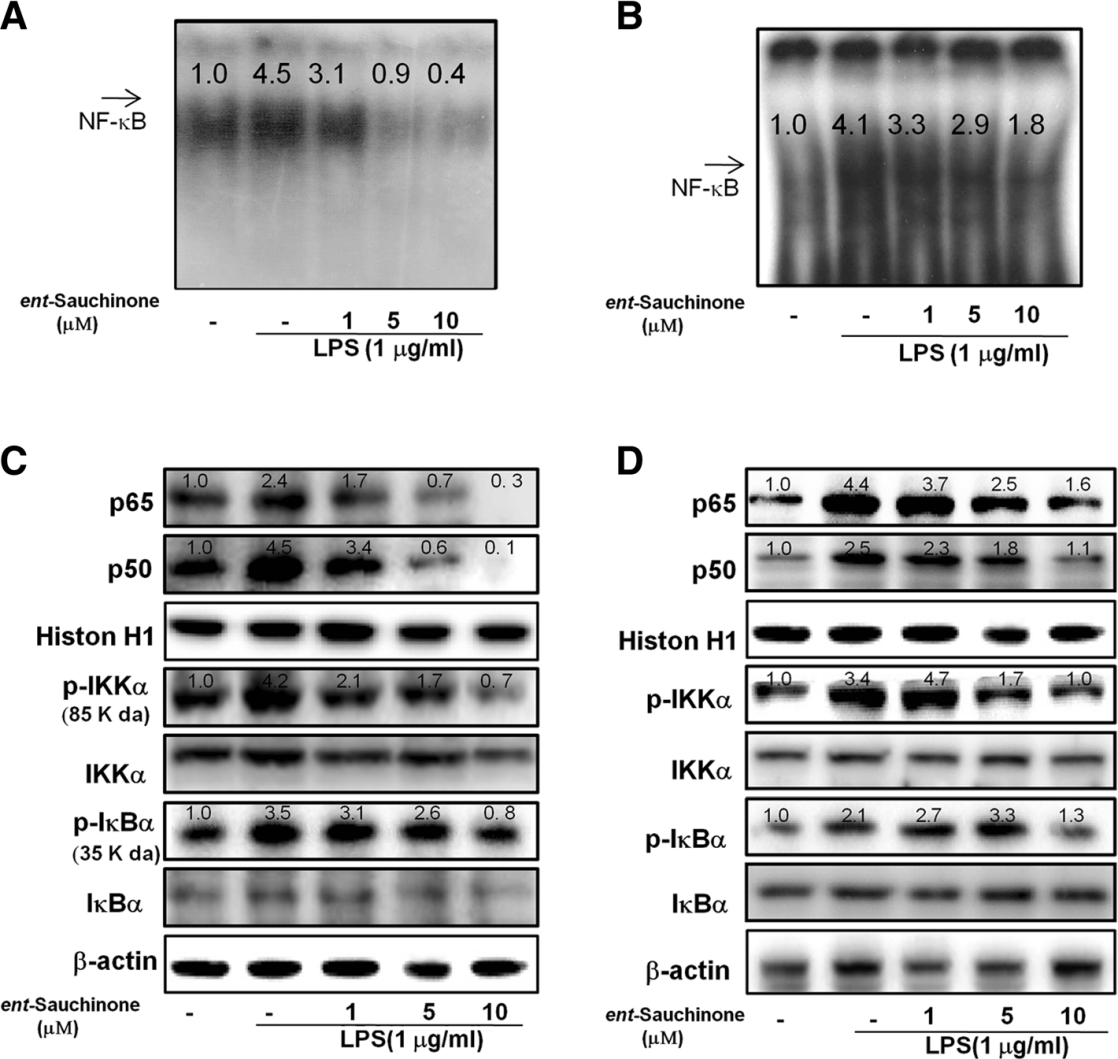
**Effects of *ent*-Sauchinone on NF-κB DNA binding activity and protein expressions of NF- κB in astrocytes and microglial BV-2 cells.** Astrocytes **(A)** and microglial BV-2 cells **(B)** were treated with 1 μg/ml of LPS alone, or with LPS plus different concentrations (1, 5, and 10 μM) of *ent*-Sauchinone at 37°C for 1 hour. Activation of NF-κB was investigated using EMSA as described in Materials and Methods. Nuclear extracts from astrocytes treated with LPS alone (1 μg/ml) or with *ent*-Sauchinone (1, 5, and 10 μM) and LPS were subjected to DNA binding reaction with ^32^P end-labeled oligonucleotide specific to NF-κB. Specific DNA binding of the NF-κB complex is indicated by an arrow. Similar results were obtained from at least three different sets of experiments. Equal amounts of nuclear extract (40 μg) were subjected to 10% SDS-PAGE. Nuclear translocation of p50 and p65, and degradation of IKKα and IκB were detected by western blotting using specific antibodies. β-Actin protein was used here as an internal control **(C and D)**. Values represent the mean ± S.E. for three independent experiments performed in triplicate, and each luciferase activity was calibrated using the amount of protein. Values above of each figure show the quantified relative expression of the proteins or DNA binding activity.

Activation of NF-κBis critical for the induction of inflammatory response genes, thus, we determined whether *ent*-Sauchinone might suppress NF-κB activation in LPS-activated BV-2 cells and astrocytes. BV-2 cells and astrocytes were co-treated with LPS and *ent*-Sauchinone for 60 minutes, which is the time needed to activate NF-κB maximally (data not shown). Nuclear extracts from co-treated cells were prepared and assayed NF-κB DNA binding activity measured by EMSA. In astrocytes cells (Figure 3A) and BV-2 cells (Figure 3B), co-treatment with *ent*-Sauchinone inhibited LPS-induced NF-κB binding activity in a concentration-dependent manner. The DNA binding activity of NF-κB was confirmed with a supershift assay with anti-p50 (Additional file 2: Figure S2A).

### Effect of *ent*-Sauchinone on LPS-induced p50/p65 translocation and degradation of IKKα and IκB

Translocation of p50 and p65 as well as IκB degradation is significant for the regulation of NF-κB. We examined the nuclear translocation of p50 and p65 by western blotting analysis. Nuclear translocation of p50 and p65 were inhibited by the co-treatment of *ent*-Sauchinone in a concentration-dependent manner in astrocytes and BV-2 cells (Figure 3C and D). Next, we found that *ent*-Sauchinone also inhibited the LPS-induced degradation of IκB. (Figure 3C and D). These results indicate that *ent*-Sauchinone inhibits the LPS-induced activation of NF-κB via an inhibition of IκB phosphorylation as well as the translocation of p50 and p65 into the nuclear. When p50 and p65 subunits were phosphorylation, they move into the nuclear.

### Effect of *ent*-Sauchinone on LPS-induced amyloidogenesis

**Figure 4:**
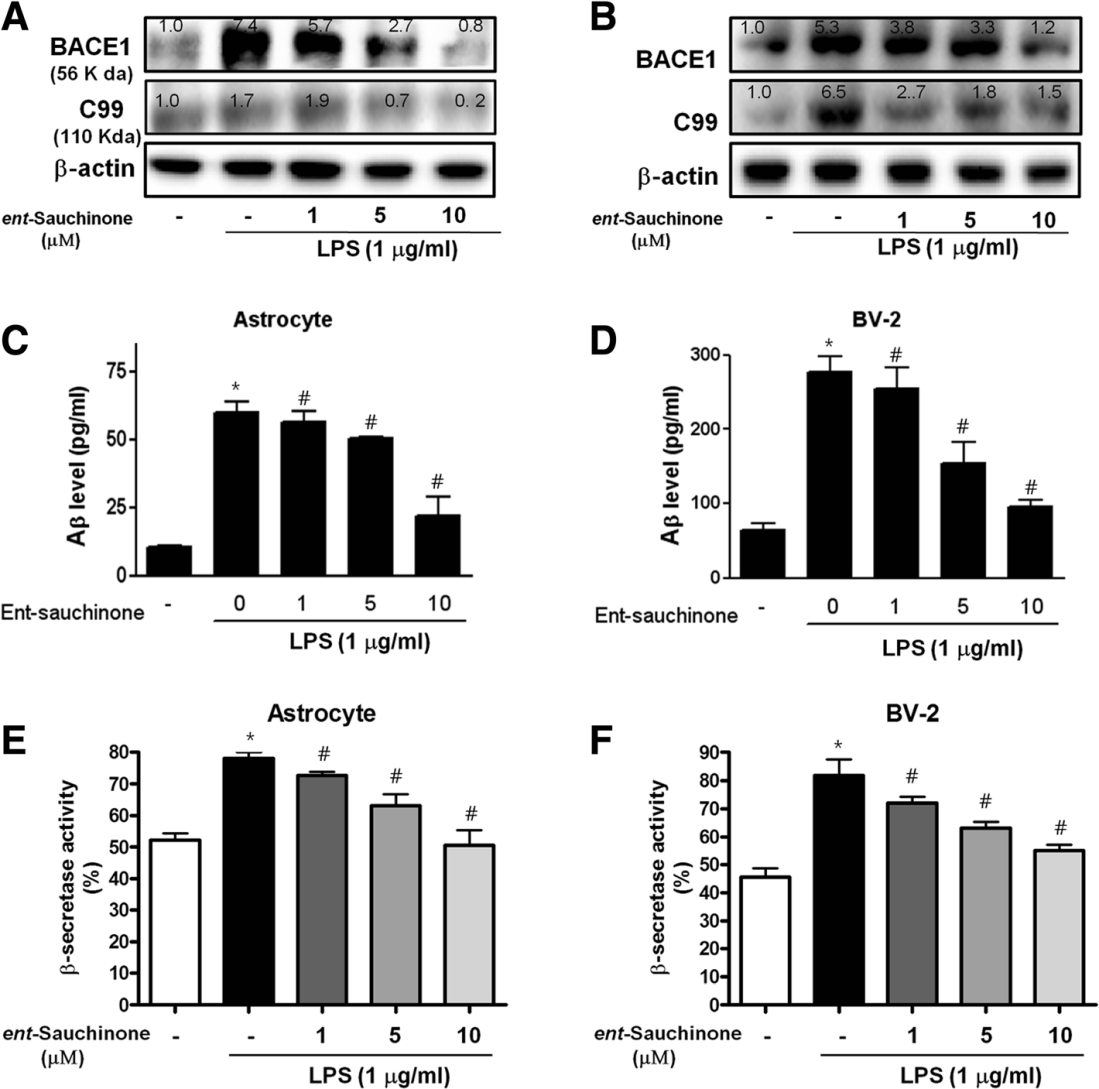
**Effect of *ent*-Sauchinone on expression of BACE1, C99, and Aβ**_**1-42**_**, β-secretase activity and Aβ**_**1-42 **_**level.** Expressions of BACE1 and C99 were detected by western blotting using specific antibodies in astrocytes (A) and microglial BV-2 cells (B). Each blot is representative of three experiments. β-Actin protein was used here as an internal control. Co-treatments with *ent*-Sauchinone and LPS for 24 hours were used. Media were collected to determine Aβ_1-42_ secretion by ELISA from astrocytes **(C)** and microglial BV-2 cells **(D)**. Values represent the mean ± S.E. of three independent experiments with triplicate. The activities of β-secretasewere assessed using commercially available assay kits as described in Materials and Methods **(E and F)**. Values represent mean ± S.E. for three independent experiments performed in triplicate. *indicates significantly different from the control group (*P* <0.05.. ^#^indicates significantly different from the LPS-treated group (*P* <0.05.. Values above of each figure mean quantified relative expression of the proteins.

The effect of inflammation on amyloidogenesis *in vitro* was also investigated since neuroinflammation can cause amyloid generation and microglia and astrocytes are a major source of neuroinflammation. Astrocytes and microglia are both mechanical and metabolic supports to neurons, regulating the environment in which they function. To determine the relationship between neuroinflammation and amyloidogenesis, we investigated whether the anti-inflammatory effect of *ent*-Sauchinone could result in anti-amyloidogenesis. As shown in (Figure 4A), when unstimulated, the cells expressed low levels of APP, site APP cleavage enzyme (BACE), and C99 protein, whereas the expressions of BACE and C99 proteins increased in response to LPS (1 μg/ml) after 24 hours. In addition, *ent*-Sauchinone also decreased LPS-induced Aβ_1–42_ secretion into the culture media of astrocytes (Figure 4C). Consistent with the expression of these proteins, activation of β-secretases, which are the rate-limiting enzymes in Aβ generation, was also increased by LPS, but inhibited by *ent*-Sauchinone in a concentration-dependent manner in astrocytes (Figure 4E and F). In microglial BV-2 cells, we also found that *ent*-Sauchinone inhibited LPS-induced expression of BACE1 and C99 (Figure 4B) as well as the Aβ level in a concentration-dependent manner (Figure 4D). Since activation of astrocytes is implicated in the activation of β-secretase, we investigated whether the numbers of activated (GFAP-positive) astrocytes and the accumulation of Aβ (Aβ_1–42_-positive cells) were concomitantly increased by LPS, and whether *ent*-Sauchinone would reduce astrocyte activation, thereby reducing Aβ-levels. To demonstrate this more clearly, cells immunoreactive for both GFAP and Aβ_1–42_ were identified using a double immunofluorescence method. The co-reactive cell number for both markers was markedly increased by LPS, but was lowered by *ent*-Sauchinone treatment (Additional file 3: Figure S3A). Moreover, to determine if treatment by *ent*-Sauchinone inhibits LPS-induced amyloidogenesis, we investigated the effects of *ent*-Sauchinone in another neuroglial cell type; microglial BV-2 cells. The co-reactive cell number for both activation of microglia (Iba1-positive cells) and Aβ accumulation (Aβ_1-42_-positive cells) was increased by LPS, but lowered by *ent*-Sauchinone treatment (Additional file 3: Figure S3B). These results further indicate that the amyloidogenic pathway can be promoted by neuro-inflammatory stimulation, and the anti-inflammatory effect of *ent*-Sauchinone can result in anti-amyloidogenesis.

### Effect of *ent*-Sauchinone on LPS-induced STAT3 activities

**Figure 5:**
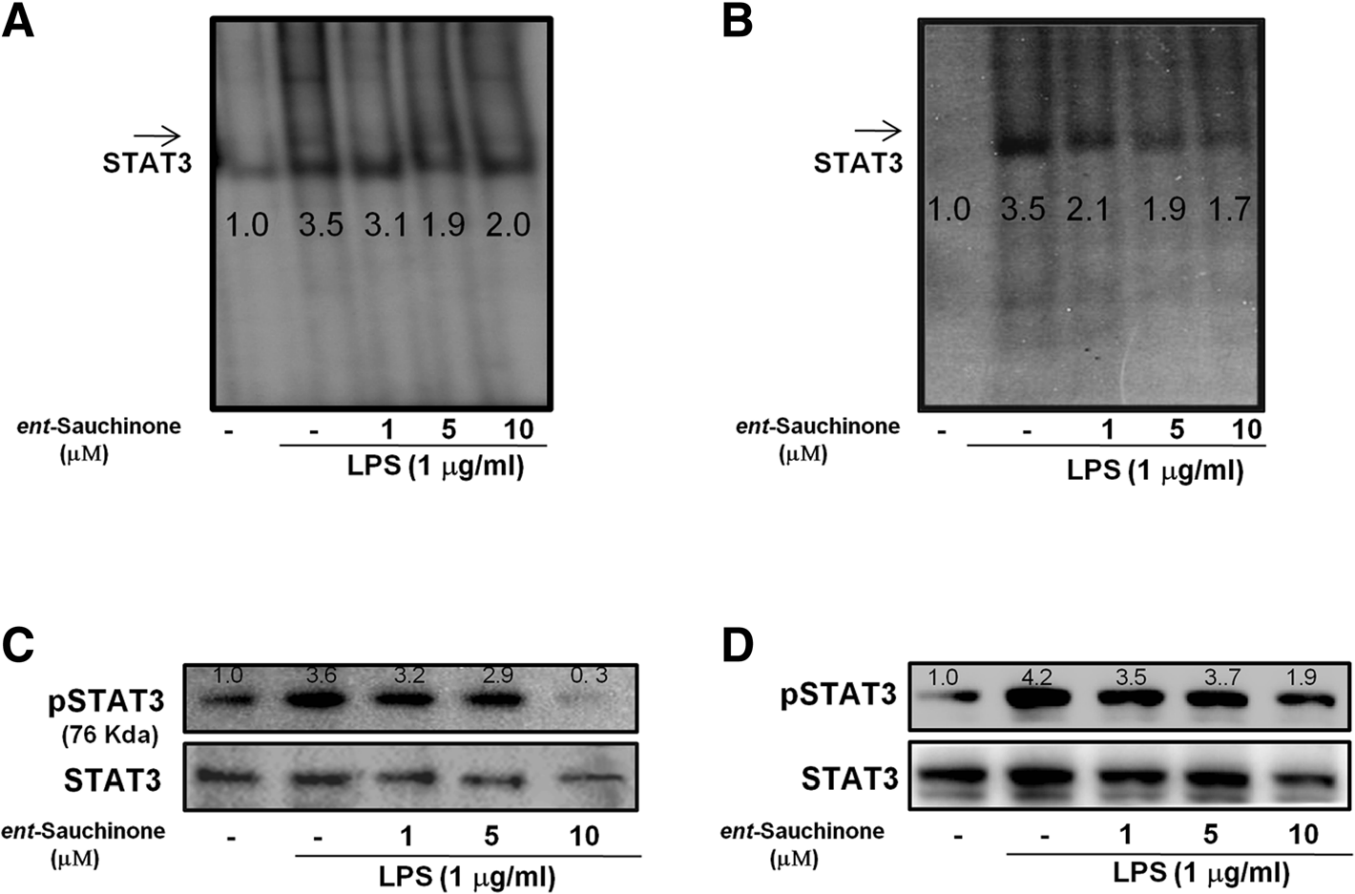
**Effects of *ent*-Sauchinone on STAT3 DNA binding activity and protein expressions of STAT3 were detected by western blotting using specific antibodies in astrocytes and microglial BV-2 cells.** Astrocytes **(A)** and microglial BV-2 cells **(B)** were treated with 1 mg/ml of LPS alone or with LPS plus different concentrations (1, 5, and 10 μM) of *ent*-Sauchinone at 37°C for 1 hour. DNA binding activity of STAT1 and STAT3 was investigated using EMSA as described in Materials and Methods. Nuclear extracts were subjected to DNA binding reaction with ^32^P end-labeled oligonucleotide specific to STAT3. Specific DNA binding of the STAT3 complex is indicated by an arrow. Similar results were treated with 1 μg/ml of LPS alone, or with LPS plus different concentrations (1, 5, and 10 μM) of *ent*-Sauchinone at 37°C for 1 hour. Equal amounts of total proteins (40 μg/lane) were subjected to 10% SDS-PAGE, and activation of STAT3 (phosphorylation) was detected by western blotting using specific antibodies in astrocytes **(C)** and in microglial BV-2 cells **(D)**. Values below or above of each figures mean quantified relative expression of the proteins or DNA binding activity.

STAT3 cooperates with NF-κB in controlling the expression of genes contributing to inflammation and amyloidogenesis. Consistent with the inhibitory effect on NF-κB activity, DNA binding activity of STAT3 elevated by LPS was significantly reduced by *ent*-Sauchinone in both astrocytes (Figure 5A) and microglial BV-2 cells (Figure 5B). Astrocytes and microglial BV-2 cells were treated with LPS (1 μg/ml) or co-treated with LPS and *ent*-Sauchinone for 24 hours. LPS-induced STAT3 activity (phosphorylation), which was markedly inhibited by the co-treatment with *ent*-Sauchinone in astrocytes and microglial BV-2 cells in a concentration-dependent manner (Figure 5C and D). The DNA binding activity of STAT3 was confirmed with a supershift assay with anti-STAT3 (Additional file 2: Figure S2A).

### Involvement of the STAT3 pathway in the inhibitory effect of *ent*-Sauchinone on LPS-induced neuroinflammation amyloidogenesis

**Figure 6:**
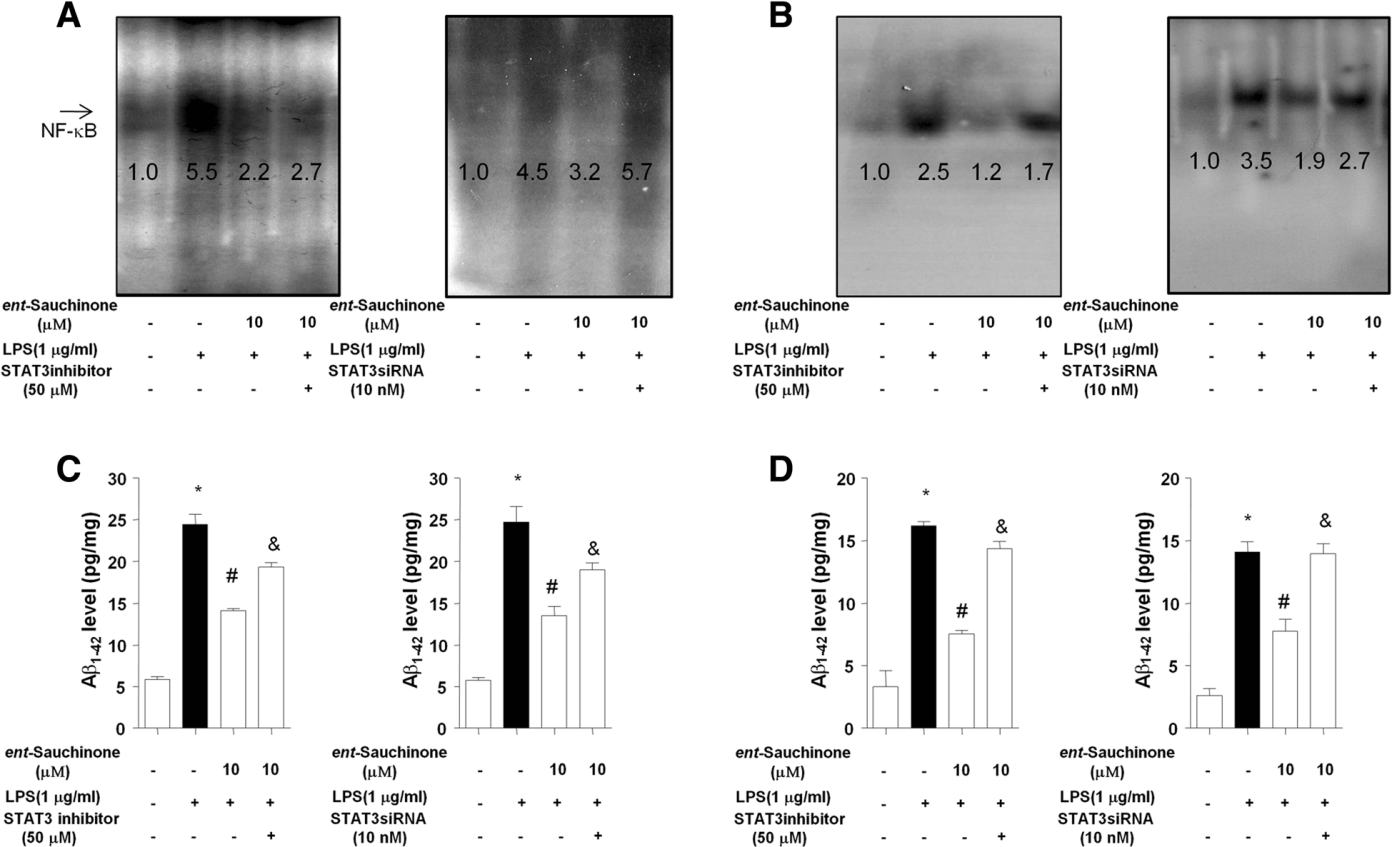
**Effects of inhibition of STAT3 on the effect of *ent*-Sauchinone on NF-κB activation and A level in cultured astrocytes and in microglial BV-2 cells.** To block the STAT3 pathway, cells were treated with the STAT3 inhibitor AG490 (50 μM) or with 50 nM siRNA for STAT3 for 1 hour prior to treatment with *ent*-Sauchinone. Abolished effect of STAT3 inhibition on the inhibitory effect of *ent*-Sauchinone on NF-κB DNA binding activity in astrocytes **(A)** and microglial BV-2 cells **(B)**, and A _1-42_ level in astrocytes **(C)** and microglial BV-2 cells **(D)**. For determination of DNA binding activity of NF-κB and Aβ_1-42_ levels, cells were treated with 1 μg/ml of LPS alone, or with LPS plus different concentrations (1, 5, and 10 μM) of *ent*-Sauchinone at 37°C for 24 hours (1 hour for NF-κB) in the absence or presence of STAT3 inhibitor or siRNA. Values are mean ± S.E. for three experiments performed in triplicate. *indicates significantly different from the control group (*P* <0.05.. ^#^indicates significantly different from the LPS-treated group (*P* <0.05.. & indicates significantly different from the LPS and *ent*-Sauchinone treated group (*P* <0.05.. Values below each figure mean quantified relative DNA binding activity.

To further examine the mechanisms regulating neuroinflammation and amyloidogenesis by STAT3 and NF-κB, we used siRNA and a pharmacological inhibitor of STAT3 in astrocytes and microglial BV-2 cells activated by LPS, and investigated the participation of the STAT3 pathway in neuroinflammation and amyloidogenesis. First, we examined the effectiveness of STAT3 siRNA on pSTAT3 expression and BACE1 in cultured microglial BV-2 cells, and found that knockdown with STAT3 siRNA completely abolished STAT3 expression as well as BACE1 expression (Additional file 2: Figure S2B). *ent*-Sauchinone inhibited Aβproduction and NF-κB activity induced by LPS treatment in astrocytes. These inhibitory effects were abolished by the downregulation of STAT3 expression with the pharmacological STAT3-specific inhibitor AG490 (50 μM) or with siRNA in cultured astrocytes (Figure 6A and C) and microglial BV-2 cells (Figure 6B and D). These findings indicate that activation of STAT3 by *ent*-Sauchinone may not only inhibit neuroinflammation but also prevent neuroinflammation-induced Aβ production in astrocytes and microglial BV-2 cells.

### The interaction between *ent*-Sauchinone and STAT3

The interaction of *ent*-Sauchinone Sepharose 6B beads with STAT3 recombinant protein or cell lysate containing STAT3 protein was assessed using a pull-down assay. The interaction of *ent*-Sauchinone Sepharose 6B beads with STAT3 was then detected by immunoblotting with the anti-STAT3 antibody. The results indicated that *ent*-Sauchinone interacted with recombinant STAT3 protein or cell lysates containing STAT3 from microglial BV-2 cells (Figure 1B). To identity the binding site of *ent*-Sauchinone to STAT3, we performed computational docking experiments with *ent*-Sauchinone and STAT3 (Figure 1C and D). The binding study performed using Autodock Vina software shows that *ent*-Sauchinone binds in a concave binding pocket of STAT3 where three domains of the DNA binding domain (DBD), the linker domain [24], and the coiled-coil domain (CCD) all meet together. The binding pocket is composed of *Gln247*, *Ala250*, *Cys251*, *Ile258*, *Arg325*, *Pro333*, and *Pro336*.

## Discussion

Astrocytes and microglial cells play a key role in regulating the inflammation of the central nervous system through the generation of NO, ROS, and other cytokines. In this study, we investigated the inhibitory effects of *ent*-Sauchinone on LPS-induced NO and ROS production and expression of iNOS and COX-2 in BV-2 cells and astrocytes. *ent*-Sauchinone (1, 5, and 10 μM) significantly inhibited LPS-induced NO and ROS production in a concentration-dependent manner. These inhibitory effects may not be related to its cytotoxic effects since no effects on cell viability were observed at concentrations of up to 10 μM in BV-2 cells and astrocytes. Comparison with the IC_50_ value of indomethacin (53.8 μM, data not shown), a well-known non-steroidal anti-inflammatory drug, in LPS-stimulated BV-2 cells and astrocytes indicates that *ent*-Sauchinone has superior effects on the inhibition of NO production [25,26]. This inhibitory effect on NO production could be related to the gene expression of iNOS and COX-2 since *ent*-Sauchinone inhibited the iNOS protein in BV-2 cells and astrocytes. These results showed that *ent*-Sauchinone could interfere with LPS-induced inflammatory signaling related to the production of pro-inflammatory molecules.

Since NF-κB is a critical transcriptional factor regulating the expression of iNOS and COX-2, we examined the effect of *ent*-Sauchinone on LPS-induced activation of NF-κB. Consistent with the inhibitory effect on iNOS and COX-2 expression, *ent*-Sauchinone inhibited NF-κB-specific DNA binding activity in a concentration-dependent manner. The promoter of the iNOS gene contains two major discrete regions synergistically functioning for the binding of transcription factors: one for NF-κB, which is mainly activated by LPS [27]. The promoter of COX-2 has also one NF-κB consensus DNA sequence. Therefore, inhibition of NF-κB activation could be significantly related to the inhibitory effect of *ent*-Sauchinone on iNOS and COX-2 expression. Several studies have shown that anti-inflammatory agents can inhibit NF-κB activity. Sesquiterpene lactones prevented the activation of NF-κB resulting in the prevention of inflammatory responses [28]. Lee *et al.* have also demonstrated that 2’-hydroxy-cinnamaldehyde deactivated NF-κB (p50) in the anti-inflammatory reaction in macrophage RAW 264.7 cells [29]. We also found that 2,4-bis(p-hydroxyphenyl)-2-butenal inhibited LPS-induced DNA binding activity of NF-κB in cultured astrocyte and microglial BV-2 cells, thereby inhibiting neuroinflammation [30]. Therefore, our data indicate that *ent*-Sauchinone inhibits the expression of iNOS, COX-2, and NO and ROS production by the inhibition of NF-κB activity, and suggest that *ent*-Sauchinone may be useful as an anti-inflammatory agent.

STATs play critical roles in the inflammatory signaling cascades triggered by inflammatory stimuli such as LPS, interferon gamma (IFNγ), and other cytokines [31]. STATs in the cytoplasm translocate into the nucleus, where they participate in the expression of many pro-inflammatory genes [32]. There are several reports that STAT and NF-κB interact with each other [33]. NF-κB and STAT regulate each other through regulation of cytokine production [24]. In our study, consistent with the inhibition of NF-κB, *ent*-Sauchinone also inhibited LPS-induced STAT3 activity in astrocytes and microglial BV-2 cells. Moreover, STAT3 inhibition by siRNA and an inhibitor reversed the inhibitory effects of *ent*-Sauchinone on LPS-induced NF-κB activity. In a docking model, as well as a pull-down assay, we found that *ent*-Sauchinone binds directly to STAT3 with a binding affinity of approximately -7.9 to -8.6 Kcal/mol. These findings indicate that inactivation of STAT3 by *ent*-Sauchinone is significant to inhibit neuroinflammation in cultured astrocytes and microglial BV-2 cells.

It is noteworthy that neuroinflammation is associated with the generation of Aβ in the brains of AD patients [34]. We and other investigators found that systemic LPS injection remarkably increases neuro-inflammatory mediated amyloidogenesis [35]. Activated astrocytes and microglia release molecules such as inflammatory cytokines, ROS, and NO, which are known to induce BACE expression, and thus stimulate Aβ regeneration [36,37]. In fact, we found that GFAP or Iba1 positive cells are reactive for Aβ_1-42_, indicating that astrocytes and microglia cells are at least responsible for Aβ generation while *ent*-Sauchinone reduced these double reactive-cell numbers. Therefore, the inhibitory effect of *ent*-Sauchinone on neuroinflammation in astrocytes and microglial BV-2 cells by LPS might be associated with the reduction of amyloidogenesis. It is also important to know that NF-κB controls the expression of APP and BACE1, which enhances Aβ formation [38], and we previously found that the activation of NF-κB contributes to the increase in β-secretase in neuronal cells *in vitro* and *in vivo*[39]. It was also reported that AD brains contain increased levels of both BACE1 and NF-κB p65, and NF-κB p65 expression leads to an increase in BACE1 promoter activity and BACE1 transcription, while knockout of NF-κB p65 decreases BACE1 gene expression in cells [40]. It was also reported that activation of STAT3, an important transcriptional factor in inflammatory responses, is required for the upregulation of BACE1 transcription during amyloidogenic processing of APP [18]. Thus, STAT3 may also play an important regulatory role during neuroinflammation-associated amyloidogenesis [41]. In this study, consistent with the inhibitory effect on NF-κB, *ent*-Sauchinone inhibited STAT3 activity. Moreover, STAT3 inhibition by siRNA or an inhibitor reversed inhibitory effects of *ent*-Sauchinone on LPS-induced Aβ generation and NF-κB activity. Similar to these findings, our previous studies showed that the inhibitory effect on NF-κB and/or STAT3 DNA binding activity of thiacremonone, 4-O-methylhonokiol, 2,4-bis(4-hydroxyphenyl)-2- butenal, 2,4-bis(4-hydroxyphenyl)-2- butenal diacetate and (-)-epigallocatechin-3-gallate (EGCG) could be critical for the anti-amyloidogenic effects of these compounds [42-47]. These results indicate that the blocking effect of *ent*-Sauchinone on NF-κB and STAT3 could contribute not only to an anti-inflammatory effect but also amyloidogenic effects. These findings indicate that inactivation of STAT3-mediated NF-κB by *ent*-Sauchinone may be significant in inhibiting neuroinflammation and amyloidogenesis in cultured astrocytes and microglial BV-2 cells.

We also found that *ent*-Sauchinone has good oral and intestinal absorption as determined by the Caco-2 cell permeability assay and human intestinal absorption (HIA) rate. Several drug-likeness predictions such as Lipinski’s, Lead-like, Comprehensive Medicinal Chemistry (CMC)-like, and MACCS Drug Data ReportMDDR-like rules indicate that this compound is suitable as a drug. *ent*-Sauchinone was also evaluated as a non-mutagenic and non-carcinogenic compound by the (Absorption, Distribution, Metabolism, and Excretion (ADME)/Toxicity) test prediction program (pre ADME version 1.0.2). Even though the efficacy and potency of *ent*-Sauchinone are similar to other compounds such as thiacremonone, 4-O-methylhonokiol, 2,4-bis(4-hydroxyphenyl)-2- butenal, 2,4-bis(4-hydroxyphenyl)-2- butenal diacetate, and (-)-epigallocatechin-3-gallate (EGCG), the drug-like pharmacological properties of *ent*-Sauchinone are superior to other compounds and it can be obtained easily from nature. Thus, we believed that this compound could be applicable for *in vivo* study. Moreover, as neuroinflammation and amyloidogenesis are critical for the development of Alzheimer’s disease (AD), we will test the possible anti-AD effect of *ent*-Sauchinone in an AD animal mice model. Taken together, these data indicate that *ent*-Sauchinone could be applicable for drug development for inflammatory neurodegenerative diseases such as AD.

## Abbreviations

Aβ: Amyloid-beta protein; AD: Alzheimer’s disease; ANOVA: Analysis of variance; APP: Amyloid precursor protein; BACE: β-secretase; CCD: Coiled-coil domain; COX-2: Cyclooxygenase-2; DCF: Dichlorofluorescein; DMEM: Dulbecco’s modified eagle’s medium; ELISA: Enzyme-linked immunosorbent assay; EMSA: Gel electromobility shift assay; FBS: fetal bovine serum; GFAP: Glial fibrillary acidic protein; IFNγ: interferon gamma; IκB: Inhibitor of κB; iNOS: Inducible nitric oxide synthase; LPS: Lipopolysaccharide; NF-κB: Nuclear factor-kappa B; NO: Nitric oxide; PVDF: Polyvinylidene difluoride; ROS: Reactive oxygen species; STAT3: Signal transducer and activator of transcription 3.

## Competing interests

The authors declare that we have no competing interests.

## Authors’ contributions

JTH and BYH designed the study and prepared the manuscript. S-Y S, YYJ, CHS and JHK performed experiments. YWH, HPL and BKH measured binding affinity (STAT3 versus *ent*-Sauchinone). BYH, HOS and SML isolated and characterized *ent*-Sauchinone. DYC and SBH discussed the study. All authors have read and approved the final version of this manuscript.

## Supplementary Material

Additional file 1: Figure S1 Effect of *ent*-Sauchinone on viability of astrocytes and microglial BV-2 cells. Cell viability was evaluated using a WST-8 assay as described in Materials and Methods. Astrocytes **(A)** and microglial BV-2 cells **(B)** were incubated with *ent*-Sauchinone (1, 5, and 10 μM) in the absence of LPS for 72 hours. Results are given as a percentage of viable cells related to untreated controls. The data represent the mean ± S.E. for three independent experiments performed in triplicate. *indicates significantly different from the control group (*P* <0.05.. ^#^indicates significantly different from the LPS-treated group (*P* <0.05.. Click here for file

Additional file 2: Figure S2 Supershift assay and effect of STAT3 siRNA on the expression of p-STAT3. **(A)** Supershift assay for NF-κB and STAT3 in the nuclear extract from cultured microglial BV-2 cells by anti-p50 and STAT3. **(B)** Effect of STAT3 siRNA on the expression of p-STAT3 and BACE1 in cultured microglial BV-2 cells. All the conditions were same as those in Additional file 1: Figure S1. Click here for file

Additional file 3: Figure S3 Expression of activation markers (GFAP for astrocytes and Iba1 for microglia) and A _1–42_ in astrocytes and in microglial BV-2 cells, observed by double-fluorescence. Confocal microscope observation was performed as described in Materials and Methods. Cultured astrocytes were incubated with anti-GFAP and anti-A _1–42_ primary antibodies **(A)**, and the microglial BV-2 cells were incubated with anti-Iba1 and anti-A _1–42_ primary antibodies **(B)**. Fluorescence was developed using Alexa 568-conjugated anti-rabbit and Alexa 488-conjugated anti-mouse secondary antibodies. Images of astrocytes double-labeled with GFAP and A _1–42_ (green) antibodies show the fluorescent antibody staining separately and merged. Images of microglial BV-2 cells double-labeled with Iba1 and A _1–42_ (green) antibodies show the fluorescent antibody staining separately and merged. Click here for file
