# Supplementary figures and images for: Inhibitory effect of ent-Sauchinone on amyloidogenesis via inhibition of STAT3-mediated NF-κB activation in cultured astrocytes and microglial BV-2 cells
Source: J Neuroinflammation. 2014 Jul 2;11:118. doi: 10.1186/1742-2094-11-118 (PMC4090659; doi:10.1186/1742-2094-11-118)

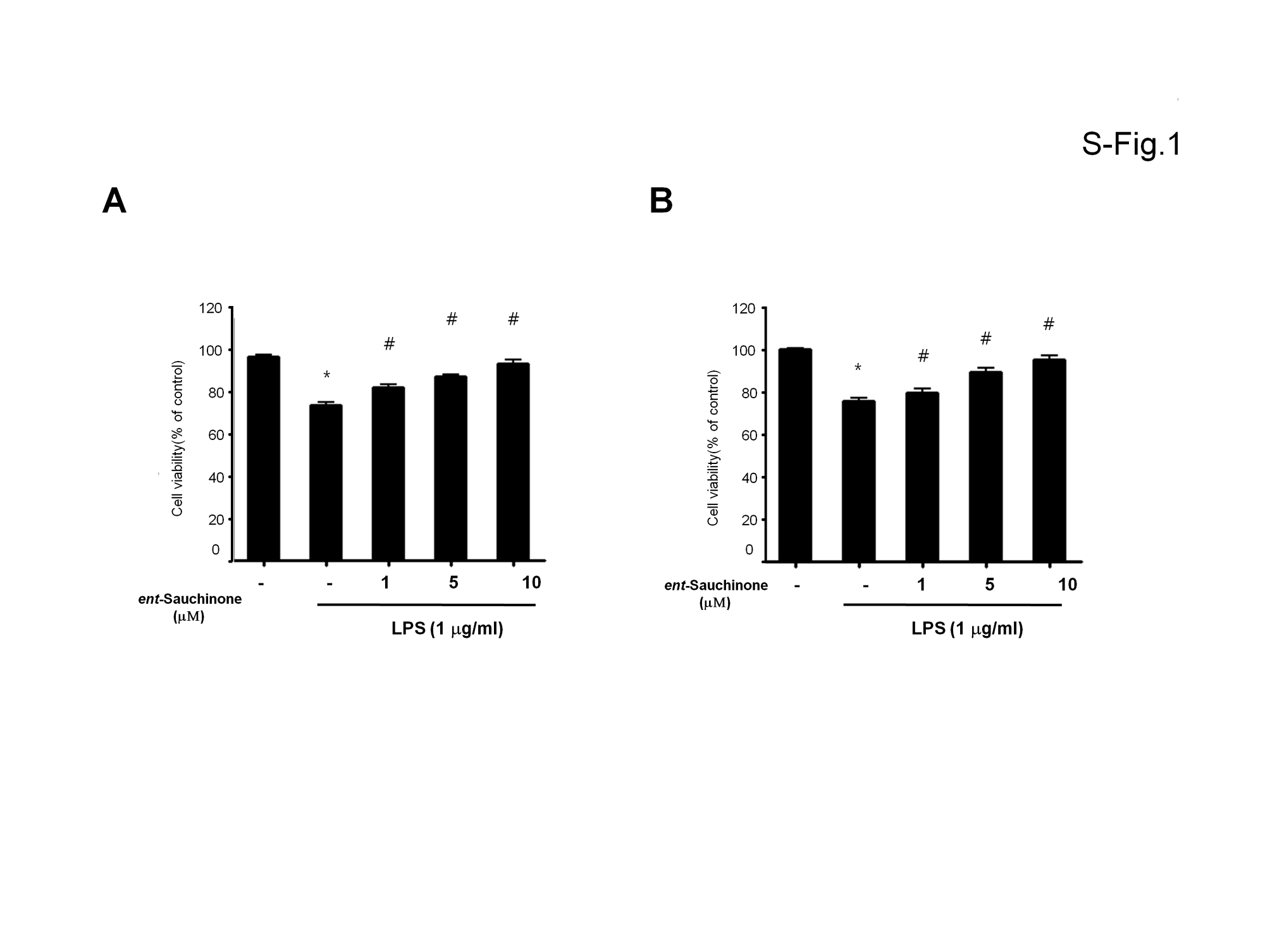

Supplement: Additional file 1: Figure S1 — Effect of ent-Sauchinone on viability of astrocytes and microglial BV-2 cells. Cell viability was evaluated using a WST-8 assay as described in Materials and Methods. Astrocytes (A) and microglial BV-2 cells (B) were incubated with ent-Sauchinone (1, 5, and 10 μM) in the absence of LPS for 72 hours. Results are given as a percentage of viable cells related to untreated controls. The data represent the mean ± S.E. for three independent experiments performed in triplicate. *indicates significantly different from the control group (P <0.05.. #indicates significantly different from the LPS-treated group (P <0.05.. [file 1742-2094-11-118-S1.tiff]

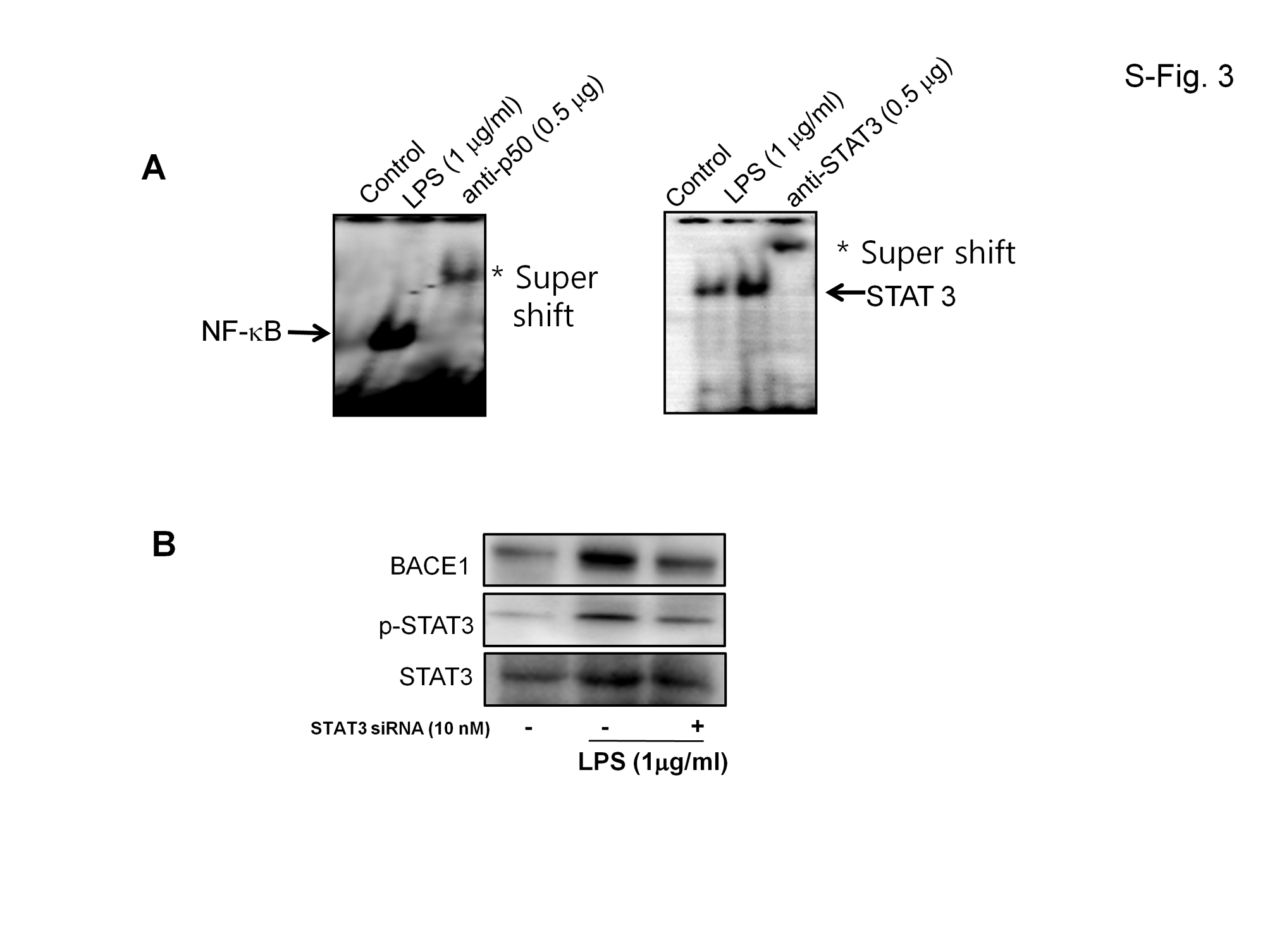

Supplement: Additional file 2: Figure S2 — Supershift assay and effect of STAT3 siRNA on the expression of p-STAT3. (A) Supershift assay for NF-κB and STAT3 in the nuclear extract from cultured microglial BV-2 cells by anti-p50 and STAT3. (B) Effect of STAT3 siRNA on the expression of p-STAT3 and BACE1 in cultured microglial BV-2 cells. All the conditions were same as those in Additional file 1: Figure S1. [file 1742-2094-11-118-S2.tiff]

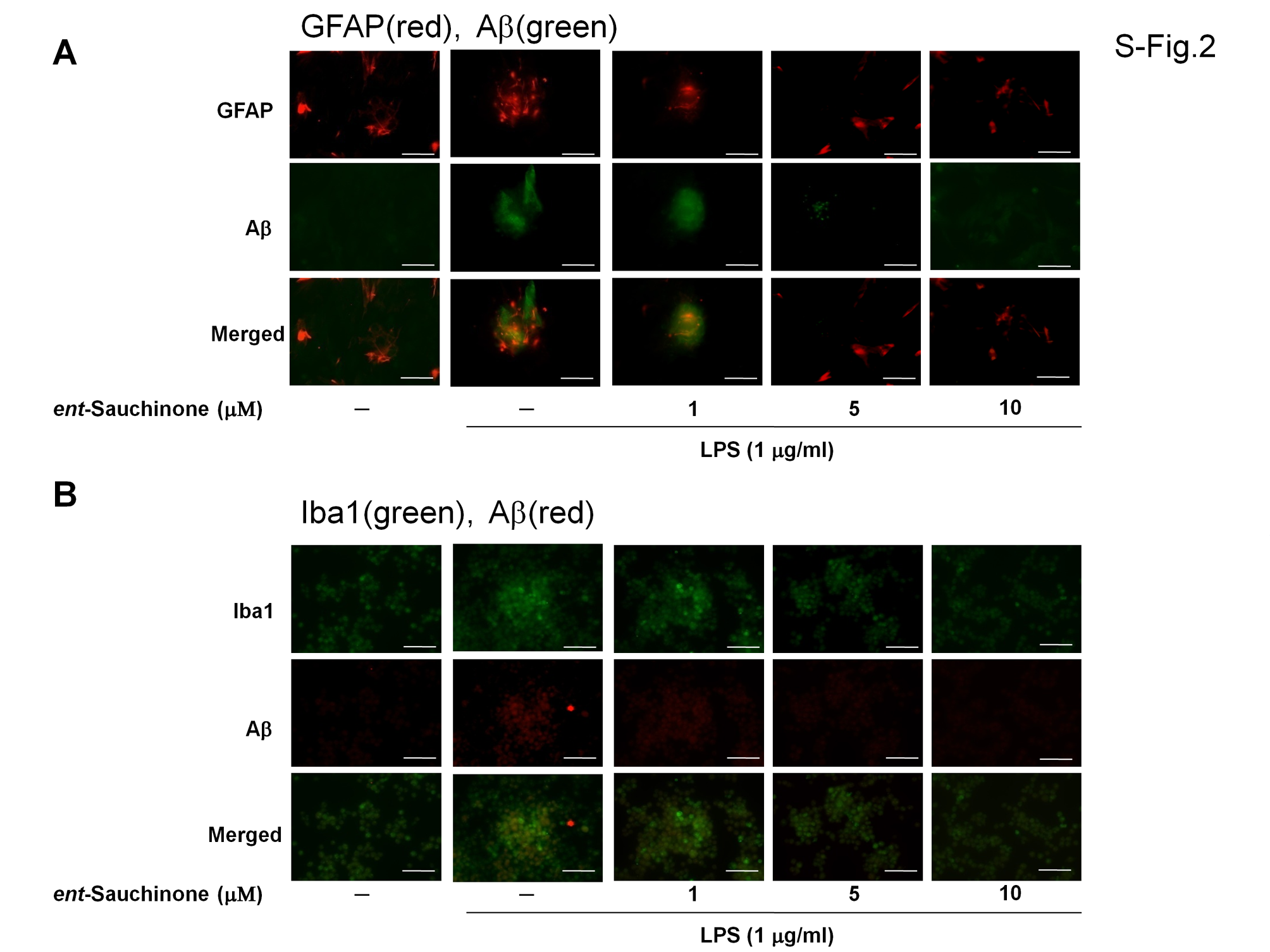

Supplement: Additional file 3: Figure S3 — Expression of activation markers (GFAP for astrocytes and Iba1 for microglia) and A 1–42 in astrocytes and in microglial BV-2 cells, observed by double-fluorescence. Confocal microscope observation was performed as described in Materials and Methods. Cultured astrocytes were incubated with anti-GFAP and anti-A 1–42 primary antibodies (A), and the microglial BV-2 cells were incubated with anti-Iba1 and anti-A 1–42 primary antibodies (B). Fluorescence was developed using Alexa 568-conjugated anti-rabbit and Alexa 488-conjugated anti-mouse secondary antibodies. Images of astrocytes double-labeled with GFAP and A 1–42 (green) antibodies show the fluorescent antibody staining separately and merged. Images of microglial BV-2 cells double-labeled with Iba1 and A 1–42 (green) antibodies show the fluorescent antibody staining separately and merged. [file 1742-2094-11-118-S3.tiff]
